# Statistical analysis in cellular systems for channel capacity improvement with dynamic pilots across different angles users

**DOI:** 10.1038/s41598-024-64288-x

**Published:** 2024-06-10

**Authors:** Shahid Ali, Nazhakaiti Yasen, Barno Sayfutdinovna Abdullaeva, Almetwally M. Mostafa, Nouf F. AlQahtani

**Affiliations:** 1 School of Electronics, Peking University, Beijing, China; 2 School of Computer Science, Communication University of china, Beijing, China; 3 Department of Mathematics and Information Technologies, Tashkent State Pedagogical University, Bunyodkor Avenue, 27, 100070 Tashkent, Uzbekistan; 4 Department of Information Systems, College Computers and Information Science, King Saud University, Riyadh, Saudi Arabia; 5 IS Department, College of Education, King Saud University, Riyadh, Saudi Arabia

**Keywords:** Frequency selective channel, Pilot shared design, Spectral efficiency, Electrical and electronic engineering, Physics

## Abstract

Accurate channel state information (CSI) is crucial for optimizing wireless communication systems. In scenarios with varying user-to-base station angles, the angle-dependent coherence time impacts conventional pilot strategies. Due to small angles, the coherence time of the user decreases dramatically because of doppler shift, which causes an increase in the number of pilots. We introduces an innovative sub-block design approach for systems with different user angles. This method harmonizes coherence time of high and low-angle users, while maintaining a constant pilot count. This not only improves spectral efficiency but also ensures accurate channel estimation. Through simulations, we demonstrate the effectiveness of our approach in enhancing both spectral efficiency upt to $10 \%$ and CSI precision. This breakthrough contributes to the advancement of channel estimation techniques in scenarios with angle-dependent coherence time, offering practical benefits to wireless communication systems.

## Introduction

“The Internet of Things (IoT) has seen substantial advancement due to robust wireless communication and computational technologies. These advancements aim to deliver comprehensive services to support intelligent transportation systems and smart cities. This is achieved through the exchange of information between public networks and vehicles at different locations^1,2^.

In terms of physical-layer design, the primary hurdle faced by the IoT is the time-frequency selective fading resulting from the rapid movement of vehicles, which change the postion angle of user to base station (BS)^3,4^ . This leads to a decrease in resource utilization efficiency, as continual deployment of pilot symbols is required for fast-varying channel estimation^5,6^.

In pursuit of attaining elevated resource efficiency while enhancing the performance of channel estimation, extensive research has been undertaken regarding the distribution of pilot symbols and the allocation of their transmit power. This inquiry is comprehensively discussed in^7–20^ and the associated references. Within the existing literature, the optimal pilot design has been ascertained for point-to-point vehicular communications, with potential applicability to multicast services involving vehicles operating at uniform locations. However, in practical Internet of Things (IoT) multicast scenarios,^21^ vehicles often exhibit disparate locations, locations essitating a tailored approach for optimizing pilot design, particularly in the context of ultra-reliable and low-latency communications, as discussed in^22,23^.

Given this context, our research endeavors to further enhance resource utilization efficiency through dynamic pilot design. This approach seeks to optimize the deployment of common pilot symbols in multicast transmissions from a base station (BS) to vehicles of varying angles at different locations.

### Related works

In the quest to enhance the efficacy of wireless communication in the presence of fading channels, the practice of pilot symbol-assisted modulation (PSAM) stands as a widely employed technique. PSAM entails the periodic insertion of known pilot symbols within the stream of unknown data. By adopting this strategy, the acquisition of accurate channel state information (CSI) becomes feasible, enabling coherent signal detection with minimal computational complexity, as discussed in^7,8^.

The placement and power allocation of pilot symbols assume a critical role in the context of channel estimation, as indicated by the references^9,10^. Within the literature, noteworthy research contributions include the work of^11,12^, where pilot design optimization was undertaken to maximize achievable data rates for block transmissions in the presence of time-frequency selective fading channels. Additionally,^13,24^ proposed a recurrent channel estimation approach, predicated on the optimal design of segmented data rates.

Furthermore, the study presented in^14,25^ delved into the analysis of pilot symbol design specifically within orthogonal frequency division multiplexing (OFDM) systems operating over doubly-selective channels. In^15,26^, the optimization of pilot design for OFDM systems was explored, particularly in scenarios involving imperfect channel state prediction.

This paper redefines multi-user hybrid massive MIMO, emphasizing angle-based orthogonality and efficient channel estimation for improved multi-user communication^27^. Lastly,^16^ introduced a pilot contamination elimination scheme tailored for multi-antenna assisted OFDM systems, with the overarching goal of reducing the training duration.

In the context of high-mobility environments, a methodical approach to channel estimation was developed in^17,28^, employing equispaced pilot symbols within a multiple-input-multiple-output (MIMO) configuration of an OFDM system. Subsequently, in^18,29^, a channel estimator with reduced computational complexity, utilizing maximum a posteriori probability principles, was introduced for mobile MIMO-OFDM systems. For vehicle-to-everything (V2X) communications in support of Internet of Vehicles (IoV) applications,^19^ proposed an optimization technique for pilot design grounded in the Markov decision process. Furthermore, in^20,30^, an investigation into interference-free pilot design was conducted within MIMO-OFDM-based V2X networks, employing zero-correlation-zone sequences.

A principal challenge encountered in V2X communications lies in the manifestation of time-frequency selective fading across physical channels, primarily stemming from the different location of vehicles. In the realm of massive MIMO-OFDM systems,^31,32^ deliberated upon the integration of both time and frequency division multiplexed pilots. In a large-scale MIMO system. In^33^ an analysis of doubly selective channel estimation, wherein a pilot pattern was devised through the insertion of guard pilots to reduce inter-carrier interference. Lastly,^34^ introduced a data-aided scheme for doubly selective channel estimation, capitalizing on an affine-precoded superimposed pilot design tailored for millimeter-wave MIMO-OFDM systems.

### Motivation and novelty

The Internet of Vehicles (IoV), established to facilitate connectivity between vehicles and roadside infrastructures, confronts numerous challenges due to the different angles of vehicles. One significant challenge is the need for doubly selective channel estimation, which necessitates a substantial allocation of pilot resources to estimate a large number of channel coefficients, particularly in the context of large-scale Multiple-Input Multiple-Output (MIMO) systems. Conversely, the escalating mobile data traffic, projected to reach 288 exabytes per month by 2027 according to Ericsson’s forecast^35^, not only demands reduced energy consumption by service providers to mitigate carbon emissions but also compels them to enhance spectral efficiency to achieve higher effective throughput.

As an efficient approach to enhance resource utilization efficiency, multicast communication has been widely employed within the IoV ecosystem. It aims to reduce energy consumption and improve spectral efficiency while ensuring the quality of service (QoS)^36,37^. Nevertheless, the challenge arises from the fact that multicast groups typically comprise multiple vehicles with different angles, resulting in substantial pilot overhead resource consumption.

In light of these challenges, we propose an innovative pilot design for IoV multicast services involving multiple vehicles with diverse angles. This approach employs common pilot symbols shared within the multicast group and dynamically tailors the design based on the varying angles of the vehicles. The primary objective is to significantly reduce the pilot overhead required for doubly selective channel estimation. Specifically, the novelty of our work is summarized in comparison with related studies on channel estimation and pilot design.

The salient advantages offered by our dynamic pilot design are outlined below:

#### Reduction of pilot overhead

In contrast to the traditional pilot pattern design, which is typically tailored for point-to-point links, our approach centers on dynamic pilot design within multicast services. By utilizing shared pilot symbols among multiple vehicles, we achieve a substantial reduction in pilot overhead.

#### Multicast to different angles vehicles

The pilot design previously proposed for point-to-point vehicular communications can find application in multicast services involving vehicles with uniform angles. However, in our current work, we optimize our dynamic pilot design specifically to accommodate multicast scenarios with vehicles of varying angles.

#### Improvement of resource utilisation efficiency

Our dynamic pilot design leads to a reduction in pilot overhead, consequently enhancing spectral efficiency by facilitating higher data rates. Additionally, it contributes to improved energy efficiency as a result of achieving higher effective throughput.

Throughout this paper, the following mathematical notations are used: Boldface uppercase and lowercase letters denote matrices and vectors, respectively. In particular, $\mathbf{{0}}_{1 \times M}$ denotes the $1 \times M$ zero vector and $\mathbf{{I}}_M$ denotes the $M \times M$ identity matrix. The transpose and the modulus operators are denoted by $(\cdot )^{\textrm{T}}$ and $|\cdot |$, respectively. The complex normal distribution with mean $\mu$ and variance $\sigma ^2$ is denoted by $\mathscr{C}\mathcal{N}(\mu ,\sigma ^2)$. The greatest integer function is denoted by $\lfloor \cdot \rfloor$.

## Preliminaries

In wireless communications, the PSAM is used for the estimation of CSI and, thus, understand the propagation property of a link. For an (*M*, *N*) flat-fading MIMO channel with *M* transmit and *N* receive antennas, the $N \times M$ random matrix $\textbf{H}=[h_{nm}]=[\textbf{h}_1, \textbf{h}_2, \cdots , \textbf{h}_{M}]$ represents the channel matrix in a channel realisation, where the $N \times 1$ vector ${\mathbf{h}}_{m} = [h_{{1m}} ,h_{{2m}} , \cdots ,h_{{Nm}} ]^{{\text{T}}}$ contains the flat-fading channel coefficients from the $m\textrm{th}$ transmit antenna to all the *N* receive antennas. Herein, all the components in $\textbf{H}$ are assumed independent and identically distributed (i.i.d.) circularly symmetric complex Gaussian random variables with zero mean and unit variance, i.e., $h_{nm} \sim \mathscr{C}\mathcal{N}(0,1)$, $m \in \{ 1, 2, \cdots , M\}$, $n \in \{ 1, 2, \cdots , N\}$.

**Figure 1:**
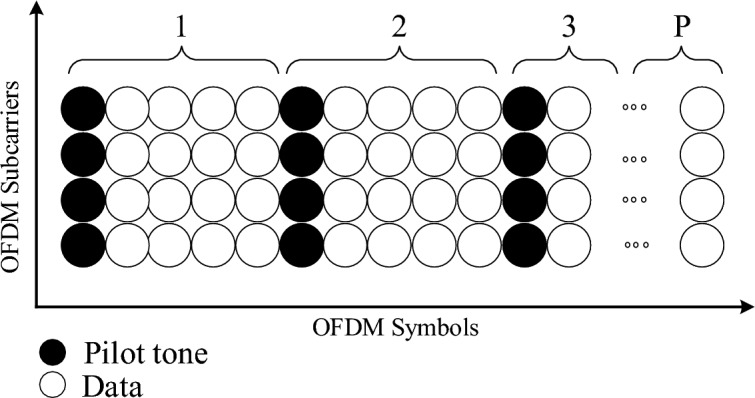
The structure of a transmitted block from an arbitrary transmit antenna.

In practice, the block-fading MIMO channel model is adopted for channel estimation, where the i.i.d. channel coefficients in a channel realisation $\textbf{H}$ are sampled from the complex Gaussian ensemble $\mathscr{C}\mathcal{N} (0,1)$ at the start of each block and remain constant for *C* symbols. This process is repeated for every block in an i.i.d. manner, and the block length is *C* in the unit of symbols. The structure of a transmitted block from an arbitrary transmit antenna, $m \in \{1, 2, \cdots , M\}$, is shown in Fig. 1. Within each block, *P* pilot symbols are evenly interspersed with the data at each transmit antenna for the purpose of channel estimation and, thus, the pilot overhead factor is defined as *P*/*C*. Note that, a total of *MP* pilot symbols are allocated in an MIMO system of *M* transmit antennas.

At the receiver, the CSI $\textbf{h}_m$, spanning from the $m\textrm{th}$ transmit antenna to all the *N* receive antennas, is estimated according to the pilot observations, $m \in \{1, 2, \cdots , M\}$, and then, the CSI estimation is used for the coherent detection of the data $\textbf{s}_p$, $p = 1, 2, \cdots , P$, where the data in a sub-block is denoted by a $1 \times (C/P-1)$ vector $\textbf{s}_p$.

The observed pilot symbols originating from the $m\textrm{th}$ transmit antenna is obtained by

1 $${\mathbf{Y}}_{{{\text{b}},m}} = {\mathbf{h}}_{m} \,{\mathbf{x}}_{{\text{b}}} + {\mathbf{W}}_{{{\text{b}},m}} ,$$

where the $N \times P$ matrices $\textbf{Y}_{\textrm{b},m}$ and $\textbf{W}_{\textrm{b},m}$ are the receiver’s observations of pilot symbols and additive white Gaussian noise (AWGN), respectively, pertaining to the $m\textrm{th}$ transmit antenna, $m \in \{1, 2, \cdots , M\}$. The $1 \times P$ vector ${\mathbf{x}}_{{\text{b}}} = [b,b, \cdots ,b]$ contains *P* pilot symbols originating from the $m\textrm{th}$ transmit antenna, which is known at the receiver.

Using minimum mean square error (MMSE) estimation, the CSI $\textbf{h}_m$ is estimated by^38,39^

2 $$\widehat{{\mathbf{h}}}_{m} = {\mathbf{x}}_{{\text{b}}}^{{\text{T}}} \left( {{\mathbf{x}}_{{\text{b}}} {\mathbf{x}}_{{\text{b}}}^{{\text{T}}} + \sigma _{W}^{2} {\mathbf{I}}_{P} } \right)^{{ - 1}} {\mathbf{y}}_{{{\text{b}},m}} ,$$

where $m = 1, 2, \cdots , M$, and $\sigma _W^2$ is the AWGN power.

The real CSI $\textbf{h}_m$ can then be expressed as

3 $$\begin{aligned} \textbf{h}_m = \hat{\textbf{h}}_m + \tilde{\textbf{h}}_m, \end{aligned}$$

where $\tilde{\textbf{h}}_m$ is the channel estimation error and its variance is the MMSE, given by

4 $$\begin{aligned} \sigma _{\tilde{\textbf{h}}_m}^2 = \frac{1}{1+P |b|^2/\sigma _W^2}. \end{aligned}$$

Apparently, the MMSE is reduced with the increase in the number of pilot symbols, *P*.

### Pilot design for time-frequency selective channels

The primary challenge encountered within the context of the IoV pertains to the time-frequency selective fading phenomenon, which is predominantly instigated by the different angle of vehicular entities.

In a MIMO system characterized by (*M*, *N*) dimensions and operating across doubly selective channels, the impulse response characterizing the time-varying channel from the $m\textrm{th}$ transmitting antenna to the $n\textrm{th}$ receiving antenna is formally denoted as $h_{nm}(t;\tau )$. In this representation, the parameter $\tau$ assumes values within the $\left[ {0,\,\tau _{{{\text{max}}}} } \right]$, where $\tau _{{{\text{max}}}}$ signifies the upper bound for delay spread arising from multipath propagation effects. Furthermore, the indices *m* and *n* are employed to distinguish between the transmit antennas, taking values from the set $m \in \{1, 2, \cdots , M\}$ and receive antennas, ranging from $n \in \{1, 2, \cdots , N\}$ respectively.

With a given sampling period denoted as $T_s$, an OFDM system comprises *Q* subcarriers, each exhibiting uniform frequency spacing, defined as $\Delta f = 1/(Q T_s)$. For effective doubly selective channel estimation, it becomes imperative to capture the variations across the *Q* frequency bases, all while accommodating distinct paths in the time domain.

Consequently, the impulse response $h_{nm}(t;\tau )$ can be effectively represented in discrete time as $h_{nm}(k;l)$, where the continuous-time parameters are discretized as follows: $t = k T_s$, and $\tau =l T_s$, with $k = 1,2,3,\cdots$ ranging from 1 onwards, and *l* taking values within the range $l = 0, 1, \cdots , L-1$.

**Figure 2:**
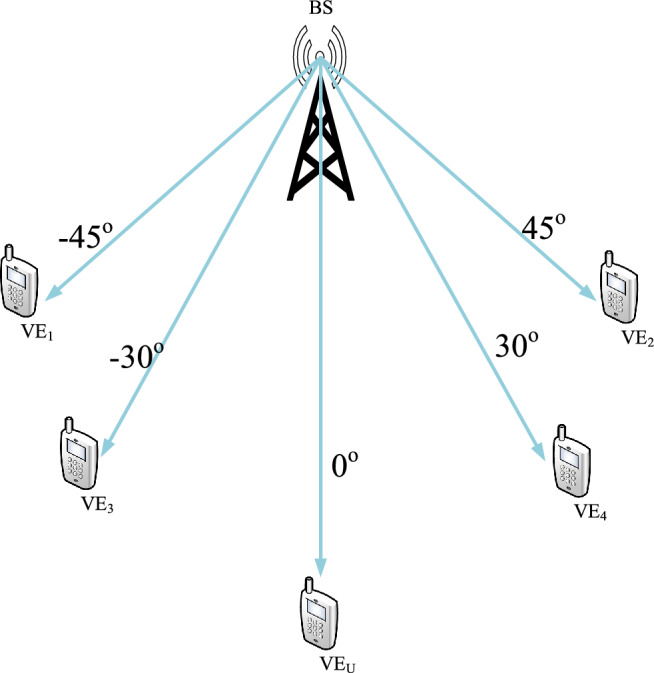
Multicast different angle VEs.

The structural configuration of a transmitted block originating from any arbitrary transmit antenna, denoted as $m \in {1, 2, \cdots , M}$, designed for the purpose of doubly selective channel estimation within a point-to-point Multiple-Input Multiple-Output (MIMO) system, is schematically illustrated in Fig. 1. Further more the VEs communicating with BS at different angle is given in Fig. 2.

In scenarios where the transmitter remains stationary while the receiver is a moving vehicle with a velocity of *v*, each block is comprised of a total of $P = \left\lfloor {f_{{\text{D}}} QT} \right\rfloor _{s} + 1$ sub-blocks. Here, $f_{{\text{D}}}$ is defined as the Doppler spread, and it can be expressed as $f_{{\text{D}}} = v\left( {f_{c} /c} \right)\,cos\theta$, where $f_{c}$ signifies the central frequency of the carrier, and the constant *c* corresponds to the angle, which is equal to $3\times 10^8$ meters per second, *v* is the constatn speed of users, and $\theta$ is the angle of user with respect to the base station.

Furthermore, the length of an individual sub-block is equal to the coherence time associated with the channel. This coherence time is determined by the equation:

5 $$\tau _{{\text{c}}} = \frac{1}{{f_{{\text{D}}} }} = \frac{c}{{\left( {vf_{c} } \right)cos\theta }},$$

This function is characterized as a monotonically decreasing one in relation to the parameter $\theta$.

For the sake of clarity and without loss of generality, let’s consider the $q\textrm{th}$ subcarrier, with $q \in {0, 1, \cdots , Q-1}$. In a sub-block associated with a specific transmit antenna, indicated by $\left\lfloor {\tau _{{\text{c}}} /T_{s} } \right\rfloor$ symbols, there exists a composition comprising a solitary pilot symbol, 2*L* zeros, and $\left\lfloor {\tau _{{\text{c}}} /T} \right\rfloor _{s} - (2L + 1)$ unknown information symbols.

More specifically, within the $p\textrm{th}$ sub-block, denoted by $p \in {1,2,\cdots , P}$, the information symbols are encapsulated within a $1 \times \left( {\left\lfloor {\tau _{{\text{c}}} /T_{s} } \right\rfloor - (2L + 1)} \right)$ vector denoted as $\textbf{s}_p^{(q)}$. The pilot symbol, represented as *b*, is flanked by two zero vectors, each being $1 \times L$. The purpose of the *L* zeros preceding the pilot symbol is to mitigate inter-symbol interference (ISI) that may affect it, while the zeros following the pilot symbol are intended to prevent ISI emanating from it.

When considering an arbitrary block for the purpose of estimating the Channel State Information (CSI) from the $m\textrm{th}$ transmit antenna to the $n\textrm{th}$ receive antenna within the $p\textrm{th}$ sub-block, specifically over the $q\textrm{th}$ subcarrier, the received pilot symbol can be formally represented as:

This expression signifies the received pilot symbol and serves as a pivotal component in the process of CSI estimation.

6 $$\begin{gathered} y_{{\text{b}}} (q;p) = H_{{nm}} (q;p)b + w_{{\text{b}}} (q;p), \hfill \\ p = 1,2, \cdots ,P,\quad q = 0,1, \cdots ,Q - 1. \hfill \\ \end{gathered}$$

where $y_{\textrm{b}}(q;p)$ and $w_{\textrm{b}}(q;p)$ are the observed pilot symbol and the received AWGN, respectively, $m \in \{1, 2, \cdots , M\}$, $n \in \{1, 2, \cdots , N\}$.

Using MMSE estimation, the estimated CSI is obtained by

7 $$\begin{aligned} \hat{H}_{nm}(q;p) = y_{\textrm{b}}(q;p)b/(|b|^2+\sigma _W^2), \end{aligned}$$

and the MMSE is $1/(1+|b|^2/\sigma _W^2)$.

In an (*M*, *N*) MIMO system, each sub-block on a specific subcarrier across all transmit antennas contains a total of $M\lfloor \tau _{\textrm{c}}/T_s \rfloor$ symbols. These symbols encompass *M* pilot symbols, 2*ML* zeros, and $M(\lfloor \tau _{\textrm{c}}/T_s \rfloor -1-2L)$ unknown information symbols, where *L* is distinct paths within the time domain..

Consequently, the overhead factor, which encompasses both pilot symbols and zero padding, is quantified as $(2L+1)/\lfloor \tau _{\textrm{c}}/T_s \rfloor$ in the context of pilot design tailored for point-to-point doubly selective channels.

## New dynamic pilot design

**Figure 3:**
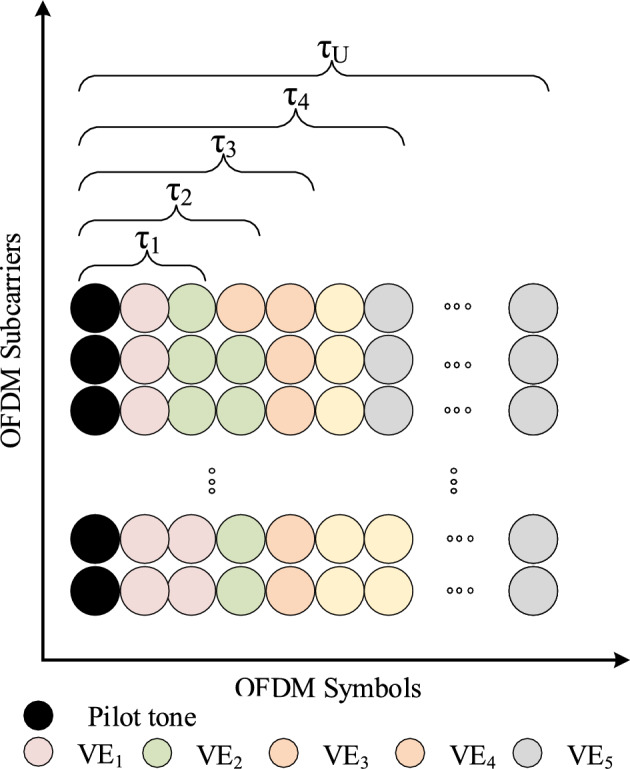
The structure of a transmitted block from an arbitrary transmit antenna with shared sub-block.

Let’s examine the multicast service as depicted in Fig. 3, where the OFDM symbol is shared among different VEs. In this scenario, there are a total of *U* vehicles within the IoV, each characterized by varying angles. These vehicles are organized in a specific order based on their angles, such that,*VE*1, *VE*2, and so forth, up to *VEU*, correspond to angles denoted as $\theta _1 \leqslant \theta _2 \leqslant \cdots \leqslant \theta _U$. Here, $\theta _u$ represents the angle of VE *u*, where *u* spans the range from 1 to *U*. At the base station, there are a total of *M* transmit antennas. For each individual VE, the doubly selective channel estimation necessitates the ability to capture variations across *Q* frequency bases within the OFDM framework.

As indicated in Eq. (5), the dimension of a sub-block within the multicast service is established by the Doppler spread induced by the differnt angles of the VEs. The Doppler spread pertaining to the channel of the $u^{\textrm{th}}$ VE, denoted as $f_u$, can be computed using the formula:

8 $$\begin{aligned} f_{u} = v( f_c/c)cos \theta _u, \end{aligned}$$

In this equation, $f_c$ represents the central frequency of the carrier employed in the multicast service, and the parameter *u* takes on values ranging from 1 to *U*.

Evidently, the coherence times associated with the channels of all *U* VEs within the multicast service follow an ordered sequence: $\tau _1\leqslant \tau _2 \leqslant \cdots \leqslant \tau _U$. Here, $\tau _u = 1/f_u$ signifies the coherence time of the channel pertaining to the $u^{\textrm{th}}$ VE. It is noteworthy that as the angle $\theta _u$ of VE *u* increases, the coherence time $\tau _u$ increases. This relationship holds true for all *u* in the range from $u \in \{1,2,3, \cdots U\}$.

One straightforward approach for designing pilot symbols for these VEs is to allocate pilot symbols individually to each of them, taking into account the coherence time of each point-to-point channel. In this manner, the overhead factor, encompassing both pilot and zero padding, within the context of doubly selective channel estimation, is determined as:

9 $$\begin{aligned} \lambda _{\textrm{con}} = \frac{U(2L+1)}{\sum _{u=1}^{U}\lfloor \tau _u/T_s \rfloor }, \end{aligned}$$

Here, $\lambda _{\textrm{con}}$ represents the overhead factor in the conventional pilot design for IoV multicast.

In order to mitigate this overhead, we introduce a dynamic pilot design within the IoV multicast framework. In this approach, common pilot symbols are shared among all *U* VEs, and the length of a multicast sub-block is standardized to $\tau _U$, which corresponds to the longest coherence time observed among all VEs within the multicast group. Consequently, this dynamic pilot design results in an overhead factor, incorporating both pilot symbols and zero padding, for the doubly selective channel estimation given by:

10 $$\begin{aligned} \lambda _{\textrm{dyn}} = \frac{2L+1}{\lfloor \tau _U/T_s \rfloor }, \end{aligned}$$

where $\lambda _{\textrm{dyn}}$ denotes the factor of overhead in our dynamic pilot design for the IoV multicast.

## Performance evaluation

In order to assess the performance and resource utilization of our dynamic pilot design, we employ key metrics, spectral efficiency,. These metrics serve to investigate the achievable data rate and effective energy consumption of our proposed approach.

To validate our analysis and design, we conducted simulations involving multicast MIMO-OFDM transmissions within a sub-block for an IoT system. The simulation parameters include:

The total number of symbols, denoted as $N = 62$. A selection of $L = 3$ for each channel. A carrier frequency of $f_c = 2.4$ GHz. A sampling period of $T_s = 36.8$ μs. The lowest three dimensional angle, $\theta _1$, set to 0. The highest dimensional angle, $\theta _U$, set to $45^{\circ }$. A total of $U = 10$ VEs. These numerical values serve as the basis for simulating and evaluating pilot overhead, spectral efficiency, and energy efficiency in our analysis and design.

**Figure 4:**
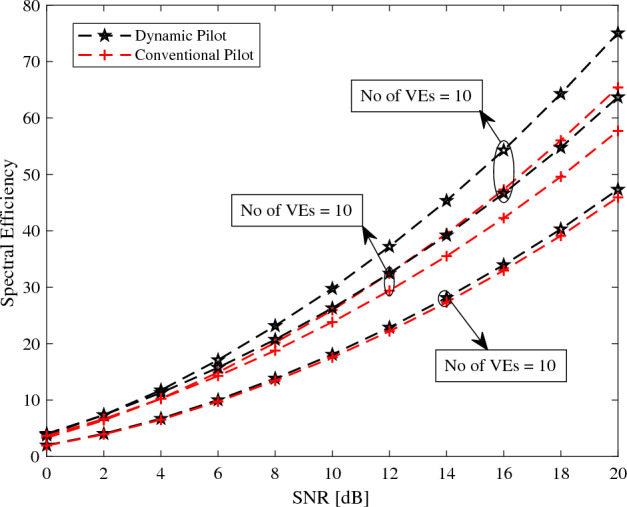
The efficiency of the system for different number of users.

**Figure 5:**
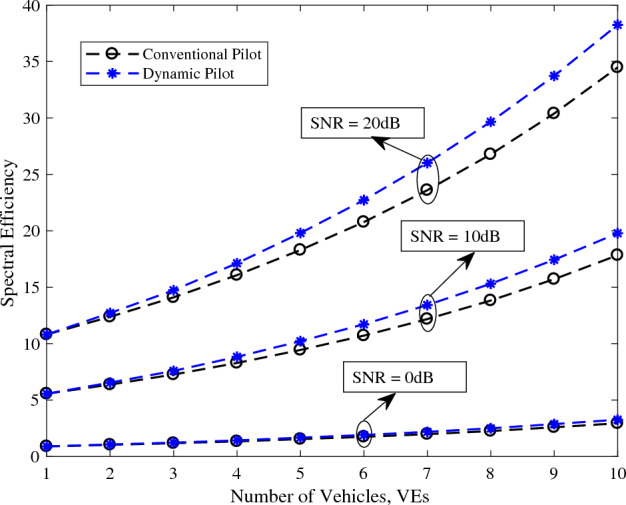
The efficiency of the system for different SNR.

To analyze the performance evolution of multicast dynamic pilot design, we define the spectral efficiency of the IoV system. Based on [26], we derived the channel throughput for multicast pilots design in MIMO-OFDM, IoV system as,

11 $$\begin{aligned} R\mathrm = (1-\lambda ) N_c E \left[ log_2(1 + \frac{\rho _s }{\sigma _\omega ^2} \textbf{H}\textbf{H}^H ) \right] , \\ \rho _s = E \left[ \Vert s^2 \Vert \right] . \nonumber \end{aligned}$$

where $\lambda$ is the pilot overhead, $N_c$ is the total number of the OFDM subcarriers in the system. The channel matrix, $\textbf{H}$ is complex $\mathbb {C}^{N\times M}$ and $\sigma _\omega ^2$ is i.i.d AWGN.

Consider the equal power equalization method for power calculation of the transmitted data of dynamic pilot design we have the channel spectral efficiency *R* given by,

12 $$\begin{aligned} R_{dyn}\mathrm = (1-\lambda _{dyn}) N_c E \left[ log_2det(\mathbf{{I}} + \frac{\rho _s }{\sigma _\omega ^2} \textbf{H}\textbf{H}^H ) \right] , \\\ . \nonumber \end{aligned}$$

here $\lambda _{dyn}$ is the pilot overhead of the multicast dynamic pilot design for doubly selective channel in IoV and *M* is the number of the transmitter antenna.

Similarly for the conventional system the throughput is given by,

13 $$\begin{aligned} R_{con}\mathrm = (1-\lambda _{con}) N_c E \left[ log_2 det(\mathbf{{I}} + \frac{\rho _s }{\sigma _\omega ^2} \textbf{H}\textbf{H}^H ) \right] , \end{aligned}$$

where $\lambda _{con}$ is the pilot overhead of conventional system. Let we have the the total number of the subcarrier $N_c = 6$, the largest angle $\theta _1 = 90$, and calculate the coherence time of the corresponding VEs based on $\theta _u = \theta _u -10(U-u)$, $u \in \{1,2, \dots , U\}$. For setting, with $L = 4$, $T_s =12.8$ μs we fetched the values of pilot overhead from Eqs. (9) and (10) in to Eqs. (12) and (13) to figure out the simulation results for spectral efficiency.

In Fig. 4, the spectral efficiency of system is simulated with SNR of 20dB. Its is considered for three different scenarios $U = 2,5,10$ VEs. The spectral efficiency in Eqs. (12) and (13) is plotted. It can be seen that with increase in the SNR the spectral efficeincy of the proposed method outperform the spectral efficiency of conventional method, because in the proposed method no extra pilot symbols were inserted.

In Fig. 5, the spectral effieceny is plotted versus the number of VEs. Multicast dynamic pilot design spectral efficiency is plotted for SNR = 0 dB, 10 dB, 20 dB and compared with the conventional pilot design spectral efficiency. It can be observed that as the number of VEs increases, the spectral efficiency of the dynamic pilot design significantly surpasses that of the conventional system. This superior performance is attributed to the proposed method’s efficient sharing of a common pilot among the VEs. Unlike conventional systems that require the transmission of additional pilot symbols as the number of VEs increases, the dynamic pilot design avoids this necessity. By maintaining the same set of pilot symbols for multiple VEs, the proposed method reduces the overhead associated with pilot transmission, thereby enhancing overall spectral efficiency. This efficient utilization of pilots not only conserves bandwidth but also improves the system’s capacity to handle a larger number of VEs without compromising performance.

## Conclusion

In conclusion, this study emphasizes the paramount importance of precise Channel State Information (CSI) for the optimization of wireless communication systems. It addresses a challenge posed by varying user-to-base station angles, where angle-dependent coherence bandwidth affects conventional pilot strategies, leading to an increased pilot overhead. To address this challenge, the research introduces an innovative sub-block design approach tailored for systems with diverse user angles. This approach harmonizes coherence bandwidths for users with both high and low angles while maintaining a consistent pilot count. As a result, this method enhances both spectral efficiency and the accuracy of channel estimation, as evidenced by simulations. This breakthrough in channel estimation techniques, particularly in scenarios with angle-dependent coherence bandwidths, holds significant promise for improving the performance and reliability of wireless communication systems. It paves the way for more efficient and effective wireless communication in real-world, dynamic environments.

## Data Availability

The data will made available on a reasonable request to the corresponding author (nyasen@qq.com (NY)).
